# Brief training in mindfulness may normalize a blunted error-related negativity in chronically depressed patients

**DOI:** 10.3758/s13415-017-0540-x

**Published:** 2017-10-03

**Authors:** Maria Fissler, Emilia Winnebeck, Titus A. Schroeter, Marie Gummbersbach, Julia M. Huntenburg, Matti Gärtner, Thorsten Barnhofer

**Affiliations:** 1 Dahlem Center for Neuroimaging of Emotions, Freie Universität Berlin, Berlin, Germany; 2 Mood Disorders Centre, University of Exeter, Sir Henry Wellcome Building for Mood Disorders Research, Perry Road, Exeter, EX4 4QG UK

**Keywords:** Major depressive disorder, Chronic depression, Error-related negativity, Mindfulness, Sustained attention

## Abstract

The error-related negativity (ERN), an evoked-potential that arises in response to the commission of errors, is an important early indicator of self-regulatory capacities. In this study we investigated whether brief mindfulness training can reverse ERN deficits in chronically depressed patients. The ERN was assessed in a sustained attention task. Chronically depressed patients (*n* = 59) showed significantly blunted expression of the ERN in frontocentral and frontal regions, relative to healthy controls (*n* = 18). Following two weeks of training, the patients (*n* = 24) in the mindfulness condition showed a significantly increased ERN magnitude in the frontal region, but there were no significant changes in patients who had received a resting control (*n* = 22). The findings suggest that brief training in mindfulness may help normalize aberrations in the ERN in chronically depressed patients, providing preliminary evidence for the responsiveness of this parameter to mental training.

Major depression tends to follow a chronic or recurrent course (Kessler et al., 2005; Kessler & Bromet, 2013). In those who have entered such a course, negative mood and symptoms persist in the absence of major life events. It is therefore of particular importance to find effective ways of changing perpetuating mechanisms and the cognitive deficits that facilitate them.

Some evidence suggests that depressed patients show such deficits already at the very earliest stages of regulatory processes cascading from the detection and signalling of discrepancies to adaptive cognition and behavior. Aberrations in error-related negativity (ERN), a potential that occurs about 50 ms after the commission of an error and is maximal above central-frontal areas (Dehaene, Posner, & Tucker, 1994; Van Veen & Carter, 2002), have been suggested to represent an endophenotype for depression and psychopathology more generally (Manoach & Agam, 2013; Olvet & Hajcak, 2008). The potential signals a need for control that serves as a call for the recruitment of neural resources for compensatory responses to errors (Botvinick, Braver, Barch, Carter, & Cohen, 2001; Holroyd & Coles, 2002; Holroyd & Yeung, 2012), and is meaningfully related to individual differences in cognitive control. In healthy individuals, larger ERN is associated with increased executive capacity and attentional control (Larson & Clayson, 2011), as well as increased self-regulatory capacities more generally such as the ability to cognitively regulate daily stress (Compton et al., 2008).

What is particularly relevant in the context of depression is that the potential is determined not only by cognitive, but also by affective and evaluative processes (Hobson, Saunders, Al-Khindi, & Inzlicht, 2014; Nelson, Jackson, Amir, & Hajcak, 2015). The ERN has been found to be sensitive to internal threat, and tasks that increase salience of errors or include incentive conditions have been demonstrated to enhance the potential (Moser, Moran, Schroder, Donnellan, & Yeung, 2013). In line with this, studies that have used tasks with error feedback or emphasized the importance of fast performance mostly found an enhanced ERN in depression (Chiu & Deldin, 2007; Holmes & Pizzagalli, 2008, 2010). However, in the absence of modulatory effects of internal threat, studies have tended to uncover deficits in ERN in depressed patients. From a biological perspective, the ERN has been suggested to arise as a consequence of the disinhibition of dopaminergic neurons in the anterior cingulate cortex (ACC) when events are worse than expected (Holroyd & Coles, 2002). Findings of blunted ERN in depression (Ladouceur et al., 2012; Schoenberg, 2014; Schrijvers et al., 2008) are consistent with evidence for deficits in phasic dopamine release in this group (Dillon et al., 2014), and correlational research has indicated links of ERN deficits with reward-related dysfunctions and symptom profiles such as anhedonia and psychomotor retardation (Bates et al., 2002; Foti et al., 2011; Schrijvers et al., 2008; Weinberg, Kotov, & Proudfit, 2015). Given evidence that reward-related abnormalities become exacerbated with increased disease burden (Hall, Milne, & MacQueen, 2014), such deficits should be particularly prevalent in patients with chronic depression. In addition to aberrations in magnitude of the ERN, research has suggested that depressed patients show differences in the distribution of the global electrical field associated with errors. In a study comparing depressed patients and healthy controls, Aarts, Vanderhasselt, Otte, Baeken, and Pourtois (2013) found that although the neural generators of the ERN were similar in both groups (primarily within the medial frontal cortex and dorsal anterior cingulate), group comparisons within these regions showed significant differences in the contributions from medial and dorsolateral prefrontal cortex regions (see Aarts & Pourtois, 2010, for similar changes in patients with high levels of anxiety).

Psychometric research indicates that when assessed in the absence of modulating influences, ERN magnitude is relatively stable over time (Meyer, Bress, & Proudfit, 2014; Weinberg & Hajcak, 2011). A treatment study in depressed patients that assessed ERN before and after treatment with antidepressants found that average ERN magnitude over the whole group remained effectively unchanged despite symptomatic improvements (Schrijvers et al., 2009). Similar results have been observed in treatment studies investigating the effects of cognitive behavioral therapy in other disorders (Hajcak, Franklin, Foa, & Simons, 2008; Riesel, Kathmann, & Endrass, 2014). However, treatments aimed at alleviating disorders may not necessarily affect the particular vulnerabilities reflected in alterations of the ERN. To change the ERN magnitude, it may be necessary to target relevant psychological processes more specifically.

A promising candidate for this purpose is mental training using mindfulness meditation. Mindfulness meditation, as taught and practiced in mindfulness-based cognitive therapy for the prevention of depression (Segal, Williams, & Teasdale, 2002) and in other mindfulness-based interventions (Kabat-Zinn, 1990), encompasses a range of different techniques. Focused attention practices, in which attention is brought to and sustained on a foreground object, such as the sensation of breathing, and reestablished whenever focus is lost, specifically train capacities for cognitive control and performance monitoring. At the same time, all mindfulness practices entail the cultivation of a particular stance toward present-moment experience, characterized by openness, friendliness, and curiosity, so that throughout the practices, the training of cognitive capacities is intertwined with the regulation of affective and motivational processes. The structure of mindfulness training thus seems to mirror the integration of these processes in the ERN and its modulation. Indeed, there is evidence for increased ERN magnitudes in meditators with relatively established practice, as compared to healthy controls (Teper & Inzlicht, 2013). However, no research has yet investigated the effects of mindfulness training on the ERN in patients suffering from depression, and particularly in patients with a chronic course of the disorder, in which the deficits are likely to have become more deeply engrained.

In the present study, we first compared the ERNs in chronically depressed patients and healthy controls, and then investigated the effects of mindfulness training as compared to a resting control condition in chronically depressed patients. We hypothesized that chronically depressed patients would show a reduced ERN relative to healthy controls, and that mindfulness training would significantly increase the ERN in patients, whereas this would not be the case in the resting control condition.

## Method

### Participants

Depressed patients were recruited through advertisements in newspapers, on the internet, and in public transport. The inclusion criteria at initial assessment were a current diagnosis of major depression, as assessed by Structured Clinical Interview for the DSM IV (First, Spitzer, Gibbon, & Williams, 2002); a lifetime history of depression with onset before age 19 and either chronic persistence of the symptoms or a history of at least three previous episodes of depression, two of which needed to have occurred during the last 2 years; self-reported severity of the current symptoms at a clinical level, as indicated by Beck Depression Inventory II (BDI II; Beck, Steer, & Brown, 1996) scores above 19; being age 25 to 60, thus excluding cases of late-onset depression; and fluency in spoken and written German. The exclusion criteria were a history of psychosis or mania, current eating disorder, obsessive–compulsive disorder, current self-harm, or current substance abuse or dependence; a history of traumatic brain injury; and current treatment with cognitive–behavioral therapy. We allowed patients who were currently taking antidepressants into the study, provided that the medication had not been changed during the last four weeks prior to entry into the study.

Healthy control participants were recruited through the same routes as patients. To be included in the control group, participants had to be free of current psychiatric disorders as assessed by Structured Clinical Interview for DSM IV and had to have a BDI II score below the threshold for minimal symptoms—that is, BDI II < 13.

### Measures

The Structured Clinical Interview for the DSM IV (SCID; First et al., 2002), a semistructured interview to determine current and past DSM-IV axis-I diagnoses, was used to assess the diagnostic status of depression at baseline and after the end of the interventions. Interviews were administered by one of two trained clinical psychologists. Lifetime course of the disorder was assessed on the basis of visual timelines using the Mondimore Scale (2007). Self-reported severity of depressive symptoms was assessed using the BDI II (Beck et al., 1996; German translation: Hautzinger, Keller, & Kuehner, 2009). Self-reported symptoms of anxious apprehension and worry were assessed using the generalized anxiety disorder 7-item scale (GAD 7; Spitzer, Kroenke, Williams, & Löwe, 2006).

### Task and materials

#### Sustained attention to response task (Robertson, Manly, Andrade, Baddeley, & Yiend, 1997)

ERN was assessed during the sustained-attention-to-response task (SART). In this task, participants are presented with a continuous array of single digits (1–9), one in each trial, and asked to withhold pressing the space bar in response to presentation of the number 3 (the target), but to press the space bar in response to all other numbers (nontargets). Participants used their dominant hand for responses and were asked to emphasize speed without sacrificing accuracy. Digits were displayed for 100 ms, followed by a fixation cross that was displayed for 1,500 ms. Following a practice block of nine trials, participants were presented with three blocks of 270 trials each, with each block initiated by the participant. Target trials were presented on 11% of the trials. Trial order was pseudo-randomized in order to avoid display of consecutive target trials. The task was administered via a PC computer using Presentation software (Neurobehavioral Systems Inc., Berkeley, CA, USA). The stimuli were presented on a 270 × 340 mm screen, positioned approximately 40 cm from the participants eyes. Digits and fixation cross were displayed in black, on a gray background.

The main behavioral outcome assessed from the task was the mean accuracy on target trials (i.e., the percentage of errors of commission on trials in which the number 3 was presented). Target errors are assumed to reflect a loss of sustained attention, since the task is performed in an automated rather than a controlled manner (Robertson et al., 1997).

### Interventions

Both interventions were delivered in a series of three 1.5-h weekly individual sessions and included intensive daily home practice. The participants in both groups received a booklet that described in detail the practices for each day, along with their rationale and related psycho-educational material. The three sessions followed a set and manualized structure. During the first session, the therapist introduced the rationale of the treatment, described relevant aspects of it, and familiarized the participant with the main practices for the coming week. The second session started with a review of experiences from the first week. The therapist addressed any questions and difficulties with the practices that had arisen during the previous week, and then introduced the main practices for the second week and their rationale. The third session served to help participants to establish ways of continuing the practices on their own following the end of the study, should they wish to do so, and also to debrief them.

#### Mindfulness training

Participants in the meditation training were asked to engage in formal meditation practice for about 25 min twice per day on six out of seven days of each week using recorded guided meditations. The practices were shorter in duration than the practices in mindfulness-based cognitive therapy (MBCT; Segal et al., 2002) in order to allow for more flexibility in scheduling the practices, but followed the standard sequence of mindfulness-based interventions, leading from practices aimed at increasing attentional control and bodily awareness to practices aimed at increasing insight and the ability to regulate negative emotions.

#### Control training

Participants allocated to the treatment control condition were asked to schedule regular rest periods as a means of deliberately retreating from the activities of the day. The length and frequency of the rest periods mirrored the time demands of the meditation training. Participants received a plausible rationale for the control training that linked acute depression to stress and suggested rest, relaxation, and disengagement from negative thinking as an initial and preliminary step toward recovery.

The treatments were delivered by trained clinical psychologists. To assess plausibility, at the end of the first session participants were asked to rate on a scale from 1 (*not at all*) to 10 (*completely*) how strongly they believed the intervention would help them. At the end of the intervention, they were asked to revisit this question and to rate the helpfulness of the intervention in hindsight. Participants recorded adherence to the daily practice on protocol sheets.

### Procedure

Potential participants were screened over the phone by the recruitment team for the main inclusion and exclusion criteria, and those likely to meet the eligibility requirements were invited to an initial assessment session, during which the Structured Clinical Interview for the DSM IV was conducted. Participants who met the inclusion criteria continued this session to fill in self-report questionnaires and then took part in the electroencephalographic (EEG) assessments. Following the pretreatment assessment, depressed participants were randomly allocated to receive either the mindfulness training or the control training. After the end of the intervention, participants took part in the posttreatment assessment session, which followed the same sequence as the pretreatment session.

Randomization was conducted following a simple randomization protocol using a computer-generated randomization sequence (permuted blocked randomization with blocks of size 4) and sealed envelopes that remained concealed until assignment to the groups.

The study protocol was approved by the ethics committee of the Charité University Medicine Berlin, Campus Mitte (EA4/055/13). All participants provided written consent prior to any research activity.

### EEG recording, data reduction, and analysis

Continuous EEG was recorded from 32 Ag/AgCl active electrode sensors with integrated noise subtraction circuits (actiCap, Brain Products GmbH, Gilching, Germany), placed according to the 10/10 system (Fp1, Fp2, F7, F3, Fz, F4, F8, FC5, FC1, FC2, FC6, T7, C3, Cz, C4, T8, TP9, CP5, CP1, CP2, CP6, TP10, P7, P3, Pz, P4, P8, PO9, O1, Oz, O2, and PO10, with the ground electrode located at AFz). Signals were recorded in a frequency range from 0.016 to 450 Hz and internally digitized with a sampling rate of 5000 Hz. Before saving to disk with a sampling rate of 1000 Hz, a digital 450-Hz anti-aliasing filter was applied (BrainAmpMR plus, Brain Products, Gilching, Germany). A common reference located at FCz was used during recording and data were re-referenced to a common average reference for offline analyses, which allowed use of FCz as electrode site for the evaluation of the ERN. Electrode impedance was maintained below 10 kΩ.

The event-related potential analysis was conducted using Brain Vision Analyzer (Version 2.0.2., Brain Products GmbH, Gilching, Germany). The data were resampled to 200 Hz and filtered with low and high cutoffs of 0.5 and 30 Hz, respectively. Vertical and horizontal eye movements were removed from the data using an automated ocular correction approach based on independent component analysis (ICA; Jung et al., 2000; as implemented in the Brain Vision Analyzer software). The vertical and horizontal components of eye movements were identified using sum-of-squared correlations with the VEOG/HEOG reference channels (in our case, Fp1 and F7) as criteria. For vertical eye movements, the components were detected by means of blink detection. For horizontal eye movements, the correlations were calculated on the basis of the entire data range. The components for which the sum of the squared correlations exceeded the percentage specified for the total value to delete (30%) were deleted from the EEG.

The EEG data were segmented into response-locked intervals, beginning 400 ms before each response and continuing for 900 ms, and then subjected to a semiautomatic artifact rejection procedure. All trials containing data that exceeded a ±100-*μ*V threshold were flagged as bad and discarded from further analyses after additional visual inspection. The remaining trials were baseline-corrected using a 200-ms window from – 400 to – 200 ms prior to response onset. To compute error response negativity, we then located the most negative peak in the – 100-ms to 100-ms window relative to response onset, on the basis of the trials on which participants had committed errors. This window was chosen to account for the fact that the ERN can begin before the recording of a motoric response. Peaks were detected automatically, and the results of the automatic peak detection were then visually inspected and corrected if necessary. Because peak measures that rely on a single data point can be easily biased by noise (Luck, 2014), particularly when the number of trial is low, we quantified the ERN as the average activity in the 100 ms around the most negative peak (i.e., 50 ms on either side of the peak; cf. Weinberg, Kotov, & Proudfit, 2015). To be included in the EEG analyses, participants had to have committed at least eight errors in the SART. This criterion was chosen on the basis of previous research that had indicated a strong relation between ERN magnitude and the number of errors in go/no go-tasks, and that this relation is attenuated from eight errors onward (Meyer, Riesel, & Proudfit, 2013). In line with this, we did not find any significant relations between numbers of errors and ERN magnitude in those with at least eight errors (see the analyses reported below). Although it is much smaller in magnitude on correct trials, a negative deflection is usually found in both error and correct trials (Burle, Roger, Allain, Vidal, & Hasbroucq, 2008), and previous investigators have therefore suggested that it is critical to examine the difference between the ERN and the correct response negativity (CRN), referred to as the ∆ERN, to assess the activity that is unique to error processing (Riesel, Weinberg, Endrass, Meyer, & Hajcak, 2013). We followed this procedure and investigated effects on both ERN amplitude and the ∆ERN. The correct response negativity (CRN) was computed using the same window and area, based on trials in which participants had provided correct responses.

## Results

### Participants

**Fig. 1:**
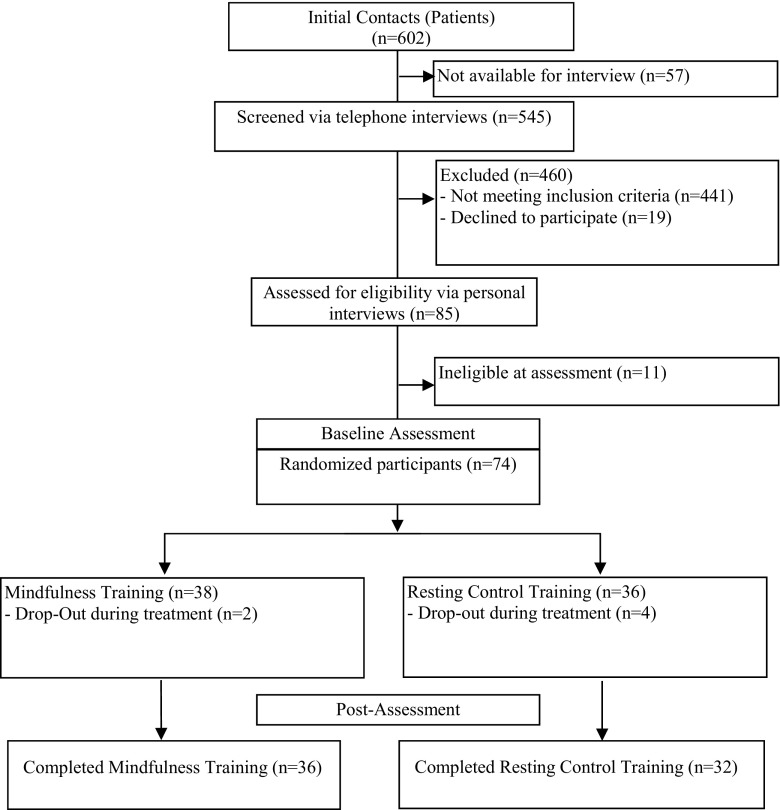
Flow of participants through the study.

Figure 1 depicts the flow of participants through the study. A total of *N* = 74 depressed participants were randomly allocated to either mindfulness training or the active control training. Of the *n* = 38 participants who received the mindfulness training, *n* = 2 dropped out of the training, leaving a sample of *n* = 36. Of the *n* = 36 participants allocated to the control condition, *n* = 4 dropped out of treatment, leaving a sample of *n* = 32. For the baseline comparisons, we recruited *n* = 25 healthy controls.

### Comparisons between depressed patients and healthy controls

Valid ERN data at baseline—that is, averaged potentials based on eight or more error trials—were available for 59 of the 74 depressed participants and 18 of the 25 healthy control participants.

#### Sociodemographic and clinical characteristics

Comparison of the depressed patients and healthy controls who committed eight or more errors, and were therefore eligible to be entered into the further analyses, indicated that the two groups were comparable in terms of gender distribution and age (see Table 1). The healthy control participants were virtually free of symptoms as assessed by the BDI II, whereas the depressed patients reported average levels of symptoms in the moderate to severe range on the BDI II, and in the mild range on the GAD 7. The group differences in self-reported symptoms were significant on both questionnaires (see Table 2).

**Table 1:** Sociodemographic characteristics and symptom levels in depressed patients (*n* = 59) and healthy controls (*n* = 18)

	Depressed	Controls	*df*	Test Statistic	*p*	Effect Size
Age, *M* (*SD*)	39.4 (12.2)	35.5 (13.2)	1, 75	*F* = 1.30	.255	*η* ^2^ = .01
Gender, *n* (% female)	36 (61)	12 (66)	1	*χ* ^2^ = .18	.665	phi = .04

**Table 2:** SART behavioral data in depressed patients (*n* = 59) and healthy controls (*n* = 18)

	Depressed	Controls	*df*	Test Statistic	*p*	Effect Size
BDI II	28.0 (7.3)	0.8 (2.1)	1, 75	*F* = 238.50	.000	*η* ^*2*^ = .11
GAD 7	9.0 (2.6)	0.3 (0.9)	1, 75	*F* = 178.49	.000	*η* ^*2*^ = .04
SART mean accuracy	.68 (.15)	.80 (.09)	1, 75	*F* = 9.78	.003	*η* ^*2*^ = .76
SART RT nontarget trials	379.4 (54.9)	352.2 (39.9)	1, 75	*F* = 3.79	.055	*η* ^*2*^ = .70

BDI II = Beck Depression Inventory II, PHQ Anxiety = Patient Health Questionnaire Anxiety Sumscore, SART = sustained-attention-to-response task, RT = reaction time.

#### Behavioral data

Analysis of the SART behavioral data showed that healthy controls had significantly higher levels of accuracy in response to target trials, whereas differences in reaction times to valid nontarget trials were on trend levels. Given that depressed patients tended to show slower responses, it seems unlikely that the group differences in accuracy were due to a speed–accuracy trade-off (see Table 2).

#### EEG analyses

**Fig. 2:**
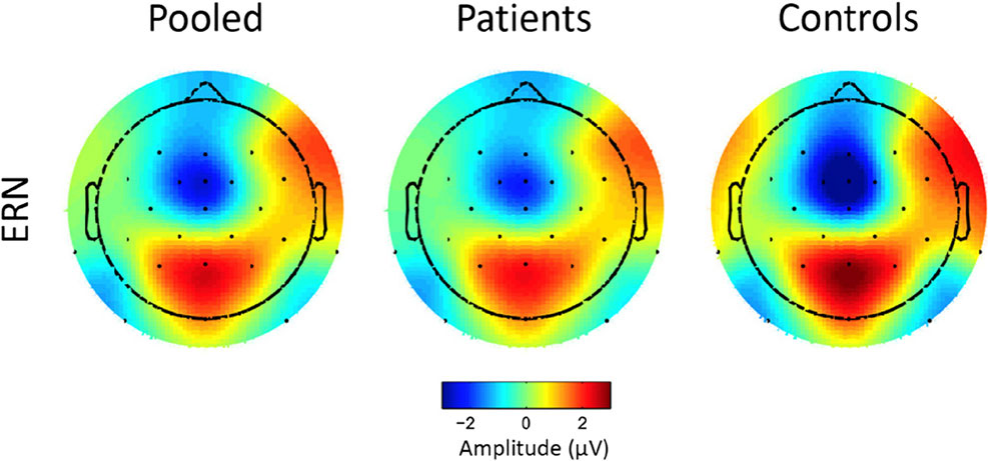
Topographical plots showing the distribution of electrical activity in error trials within a fixed time window from – 50 to 50 ms around stimulus onset in depressed patients (*n* = 59) and healthy controls (*n* = 18), as well as in the pooled sample.

Valid EEG data were available for *M* = 26.2 (*SD* = 12.7) error trials in the depressed group and *M* = 16.3 (*SD* = 7.8) error trials in the control group, *F*(1, 76) = 9.79, *p* = .003, *η* ^2^ = .12. Visual inspection of topographical plots showing the distribution of electrical activity in error trials within a fixed time window from – 50 to 50 ms around stimulus onset in the two groups indicated that, as is typical, the ERN was maximal in frontocentral regions in both groups (see Fig. 2). We therefore report analyses focused on the electrode site FCz. Visual inspection also suggested differences in topography between the two groups, with more pronounced frontal contributions in the control group. Given that earlier reports suggesting differences in the global electrical field associated with errors in depressed patients and healthy controls had implicated this region in particular (Aarts et al., 2013), we also explored the effects at electrode site Fz.

**Fig. 3:**
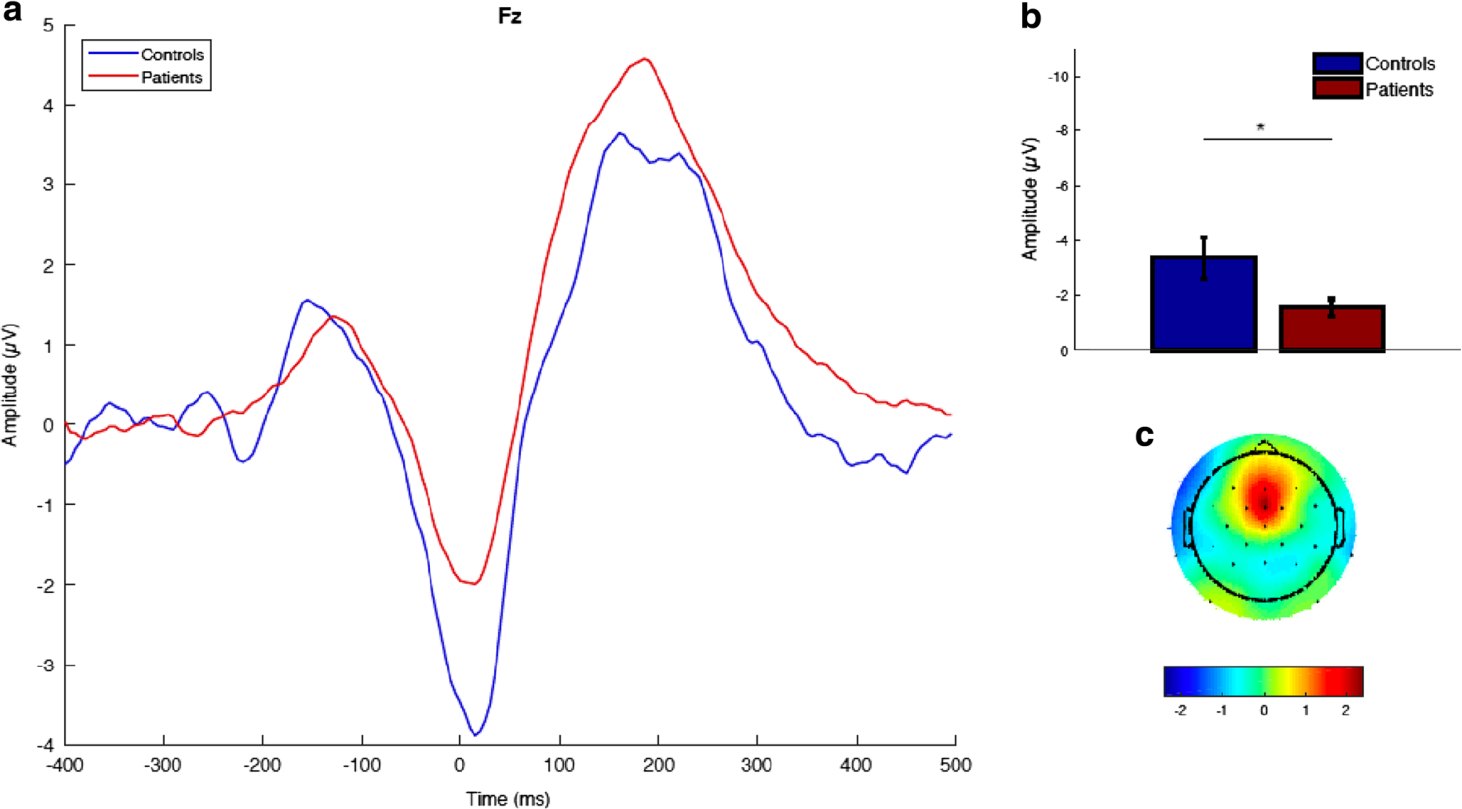
ERN differences between depressed patients and healthy controls at FCz and Fz. For each site: (Left) Time course of the ERN (grand average) in the patient and control groups. (Top right) Group differences in ERN magnitude. (Bottom right) Topographic distribution of the group differences (patients – controls).

A series of multivariate analyses of variance (ANOVAs) of ERN, CRN, and ∆ERN magnitudes indicated that, as compared to the depressed group, healthy controls had significantly higher ERN and ∆ERN magnitudes at both FCz and Fz. The two groups did not differ significantly in CRN magnitude at either of the two electrode sites (see Table 3). Grand averages and topographic maps of the ERN area measure centered around the peaks at FCz and Fz in both groups are depicted in Fig. 3. The differences in ERN magnitude and ∆ERN remained significant when the numbers of errors committed in the task were entered as a covariate, *p* < .02. When we investigated magnitude changes in mixed-model ANOVAs with Electrode Site (FCz vs. Fz) as a within-subjects factor and Group as a between-subjects factor, no significant Group × Electrode Site interactions emerged: *F*(1, 75) = 0.44, *p* = .505, *η* ^2^ = .00, for ERN, and *F*(1, 75) = 1.85, *p* = .177, *η* ^2^ = .02, for ∆ERN.

**Table 3:** Means and standard deviations of ERN and ∆ERN magnitude in depressed patients (*n* = 59) and healthy controls (*n* = 18) together with test statistics from univariate ANOVAs

	Depressed	Controls	*df*	*F*	*p*	*η* ^*2*^
	*M* (*SD*)	*M* (*SD*)				
ERN						
FCz	– 2.52 (2.91)	– 4.66 (3.94)	2, 74	6.25	.015	.07
Fz	– 1.56 (2.36)	– 3.37 (3.12)	2, 74	6.88	.011	.08
CRN						
FCz	– 0.83 (1.54)	– 0.81 (2.21)	2, 74	0.00	.965	.00
Fz	– 1.48 (1.68)	– 1.83 (2.46)	2, 74	0.46	.500	.00
∆ERN						
FCz	– 1.68 (2.69)	– 3.84 (3.28)	2, 74	8.01	.006	.09
Fz	– 0.07 (2.15)	– 1.53 (2.52)	2, 74	5.84	.018	.07

ERN = error-related negativity, CRN = correct response negativity, ∆ERN = difference between error-response negativity and correct-response negativity.

Correlational analyses within the two groups did not show any relations between SART mean accuracy and either ERN or ∆ERN magnitude, all *p*s > .10. Similarly, ERN and ∆ERN magnitudes were not significantly related to GAD-7 scores or to the GAD-7 worry items, nor were there any significant relations between ERN and ∆ERN magnitudes and BDI-II scores or the BDI-II anhedonia item, all *p*s > .10.

### Pre- to posttreatment changes in chronically depressed patients

Analyses of pre- to posttreatment changes were based on participants who completed the treatment and produced sufficient numbers of errors in the SART task to produce valid ERN data. Twenty-four of the 36 participants who completed the mindfulness training and 22 of the 32 participants who completed the resting control training had valid ERN data (eight or more errors). The final groups did not differ significantly in sociodemographic characteristics or course characteristics, including gender distribution, age, age at onset of the disorder, number of episodes of depression, and number of those using antidepressants (see Table 4).

**Table 4:** Sociodemographic characteristics, course characteristics and current use of antidepressants in depressed participants with valid ERN data who completed the mindfulness training (*n* = 24) and depressed participants with valid ERN data who completed the resting control training (*n* = 22)

Characteristic	Mindfulness Training	Resting Control Training	*df*	Test Statistic	*p*	Effect Size
Age, *M* (*SD*)	37.6 (12.5)	38.4 (12.1)	1, 44	*F* = 0.04	.832	*η* ^2^ = .00
Gender, *n* (% female)	15 (62)	13 (59)	1	*χ* ^2^(1) = 0.01	.813	*phi* = – .03
Age of onset, *M* (*SD*)	17.0 (6.5)	18.5 (12.3)	1, 66	*F* = 0.25	.615	*η* ^2^ = .01
Number of previous episodes, Med [range]	6.5 [1, 12]	6.0 [2, 35]		Median test	.526	
Current use of antidepressants, *n* (%)	4 (16)	7 (33)	1	*χ* ^2^(1) = 1.69	.193	*phi* = .19

Participants in the mindfulness training reported having completed an average of 92% (*SD* = 9.98) of the formal practices, and participants in the resting control reported having completed an average of 93% (*SD* = 7.92) of the formal practices, *F*(1, 43) = 0.24, *p* = .622, *η* ^2^ = .00, *M*_*i-j*_ = 1.34, *SE* = 0.62, 95% CI [– 6.82, 4.12]. Participants’ ratings indicated that the mindfulness intervention was perceived as being more plausible both at the beginning [mindfulness group: *M* = 8.06, *SD* = 0.99; active control group: *M* = 6.75, *SD* = 1.91; *F*(1, 36) = 6.70, *p* = .014, *η* ^2^ = .00] and at the end [mindfulness group: *M* = 9.15, *SD* = 1.18; active control group: *M* = 6.86, *SD* = 2.70; *F*(1, 39) = 12.13, *p* = .001, *η* ^2^ = .23] of the intervention period.

#### Symptoms and cognitive characteristics

At baseline, there were no significant group differences in levels of depression as measured by the BDI II, *F*(1, 44) = 1.2, *p* = .279, *η* ^2^ = .02, and in levels of anxious apprehension as measured by the GAD 7, *F*(1, 44) = 0.0, *p* = .936, *η* ^2^ = .00.

Pre-to-post changes in self-reported depression and anxiety were analyzed using repeated measures ANOVAs with Time as a within-subjects factor and Group as a between-subjects factor. The analysis of changes in BDI-II scores yielded significant main effects of both time, *F*(1, 44) = 74.2, *p* = .000, *η* ^2^ = .62, and treatment, *F*(1, 44) = 13.1, *p* = .001, *η* ^2^ = .23, that were qualified by a significant Time × Treatment interaction, *F*(1, 44) = 6.3, *p* = .016, *η* ^2^ = .12, due to stronger decreases in the mindfulness group, *M*_*i-j*_ = 16.1, *SE* = 2.0, *p* = .000, 95% CI [12.0, 20.1], than in the resting control group, *M*_*i-j*_ = 8.8, *SE* = 2.0, *p* = .000, 95% CI [4.6, 13.0]. Analysis of the GAD-7 scores yielded a main effect of time, *F*(1, 43) = 15.4, *p* = .000, *η* ^2^ = .26, but no significant effect for treatment, and no significant Time × Treatment interaction, all *p*s > .10 (see Table 5 for the means and standard deviations).

**Table 5:** Means and standard deviations of scores in self-report measures of symptoms and cognitive variables at pre- and postassessment in the mindfulness and resting control groups

	Mindfulness		Resting Control	
	Pre	Post	Pre	Post
BDI II	26.1 (6.4)	10.0 (6.8)	28.2 (6.5)	19.4 (9.0)
GAD 7	8.8 (2.1)	6.3 (3.3)	8.7 (2.9)	7.5 (3.0)
SART mean accuracy	.65 (.14)	.69 (.14)	.66 (.15)	.71 (.14)
SART RT nontarget trials	373.0 (47.5)	363.2 (41.2)	386.2 (70.7)	379.0 (58.6)

BDI II = Beck Depression Inventory II, PHQ Anxiety = Patient Health Questionnaire Anxiety Sumscore, SART = sustained-attention-to-response task, RT = reaction time.

#### Behavioral data

A mixed-model ANOVA of SART mean accuracy scores yielded a significant main effect of time, *F*(1, 44) = 4.46, *p* = .040, *η* ^2^ = .09, but no significant main effect of group, and no significant interaction of time and group, all *p*s > .20, indicating comparable improvements over time in both groups. Analysis of the SART mean reaction times in valid nontarget trials yielded no significant effects, all *p*s > .10 (see Table 5 for the means and standard deviations).

### EEG analysis

At pretreatment, valid EEG data were available for *M* = 28.5 (*SD* = 12.5) error trials in the mindfulness group and *M* = 27.5 (*SD* = 12.8) error trials in the resting control group; at posttreatment, valid EEG data were available for *M* = 25.4 (*SD* = 15.4) error trials in the mindfulness group and *M* = 23.9 (*SD* = 12.2) error trials in the resting control group (main effects and interaction, all *p*s > .05).

#### ERN

**Fig. 4:**
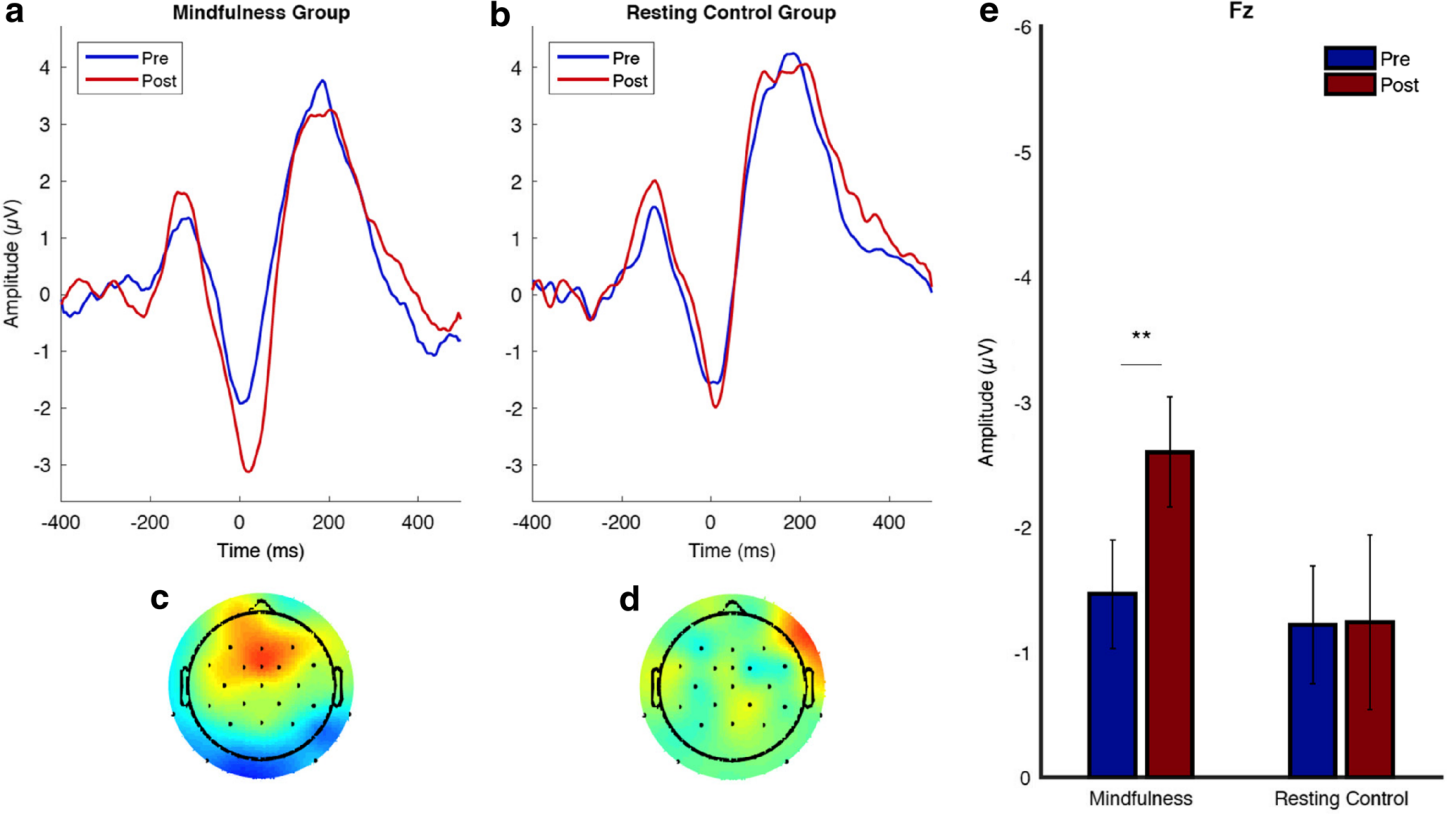
Pre- to posttreatment differences of the ERN in the mindfulness group and the resting control group at FCz and Fz*.* For each site, graphs show the time courses (grand averages) of the ERN in the mindfulness group (left) and the resting control group (middle) at the pre- and posttreatment assessments. The right panels show means and standard errors of the individual pre- and posttreatment ERN magnitudes for the mindfulness and resting control groups. The topographic maps show the distributions of pre- to posttreatment differences (pre – post) in the mindfulness group (left) and the resting control group (middle).

A mixed-model ANOVA of ERN magnitudes at FCz, with Time as a within-subjects factor and Group as a between-subjects factor, yielded a significant main effect of time, *F*(1, 44) = 5.08, *p* = .029, *η* ^2^ = .10, due to increases in the ERN over both groups, *M*_*i-j*_ = – 0.64, *SE* = 0.28, *p* = .029, 95% CI [0.06, 1.22], whereas the main effect of treatment, *F*(1, 44) = 0.002, *p* = .965, *η* ^2^ = .000, and the Time × Treatment interaction, *F*(1, 44) = 2.21, *p* = .144, *η* ^2^ = .04, did not reach significance. Analysis of the ERN magnitudes at Fz, showed a significant main effect of time, *F*(1, 44) = 4.14, *p* = .048, *η* ^2^ = .08, that was qualified by a significant Time × Treatment interaction, *F*(1, 44) = 4.15, *p* = .048, *η* ^2^ = .08, but there was no significant main effect of treatment, *F*(1, 44) = 1.56, *p* = .21, *η* ^2^ = .03. Post-hoc comparisons indicated that the Time × Group interaction was due to significant increases in ERN magnitude in the mindfulness group, *M*_*i-j*_ = – 1.17, *SE* = 0.39, *p* = .005, 95% CI [0.37, 1.97], as compared to no significant change in the control group, *M*_*i-j*_ = 0.000, *SE* = 0.41, *p* = .999, 95% CI [– 0.83, 0.83]. Figure 4 displays the grand averages and topographic maps in both groups. To test whether the interaction effects differed significantly between the two electrode sites, we conducted a mixed-model ANOVA of ERN magnitudes, with Electrode Site (FCz vs. Fz) and Time as within-subjects factors and Treatment as a between-subjects factor. This did not yield a significant Electrode Site × Time × Treatment interaction, *F*(1, 44) = 0.35, *p* = .555, *η* ^2^ = .00.

#### CRN

Analyses of the CRN magnitudes did not yield any main or interaction effects at either FCz or Fz, all *p*s > .10, apart from a marginally significant main effect of time at Fz, *F*(1, 44) = 3.04, *p* = .088, *η* ^2^ = .06, due to a decreased CRN at this site at the end of treatment, *M*_*i-j*_ = – 0.32, *SE* = 0.18, *p* = .088, 95% CI [– 0.69, 0.05].

#### ∆*ERN*

The results from analyses of ∆ERN magnitude mirrored those of ERN magnitude. A mixed-model ANOVA of ∆ERN magnitudes at FCz yielded a significant main effect of time, *F*(1, 44) = 8.96, *p* = .005, *η* ^2^ = .16, due to significant increases in ERN, *M*_*i-j*_ = – 0.86, *SE* = 0.29, *p* = .005, 95% CI [0.28, 1.45], but no significant effect of the Time × Treatment interaction, *F*(1, 44) = 1.20, *p* = .279, *η* ^2^ = .02, and no significant main effect of treatment, *F*(1, 44) = 0.25, *p* = .615, *η* ^2^ = .00. The analysis of ∆ERN magnitudes at Fz showed a main effect of time, *F*(1, 44) = 13.19, *p* = .001, *η* ^2^ = .23, that was qualified by a significant Time × Treatment interaction, *F*(1, 44) = 4.29, *p* = .044, *η* ^2^ = .08, and no significant main effect of treatment, *F*(1, 44) = 0.48, *p* = .490, *η* ^2^ = .01. Post-hoc comparisons indicated that the Time × Group interaction was due to significant increases in ∆ERN magnitude in the mindfulness group, *M*_*i-j*_ = – 1.40, *SE* = 0.34, *p* = .000, 95% CI [0.72, 2.09], but no significant change in the control group, *M*_*i-j*_ = – 0.38, *SE* = 0.286, *p* = .28, 95% CI [– 1.10, 0.33]. A mixed-model ANOVA of ∆ERN magnitudes with Electrode Site (FCz vs. Fz) and Time as within-subjects factors and Treatment as a between-subjects factor, to test whether the interactions differed significantly at the two sites, did not yield a significant Electrode Site × Time × Treatment interaction, *F*(1, 44) = 0.74, *p* = .392, *η* ^2^ = .01.

The mean ERN, CRN, and ∆ERN magnitudes and standard deviations are listed in Table 6.

**Table 6:** Mean ERN, CRN, and ∆ERN magnitude and standard deviations at pre- and postassessment in the mindfulness and resting control groups

	Mindfulness		Resting Control	
	Pre	Post	Pre	Post
ERN				
FCz	– 2.08 (2.29)	– 3.16 (2.33)	– 2.54 (3.02)	– 2.76 (2.83)
Fz	– 1.47 (2.13)	– 2.65 (2.16)	– 1.22 (2.22)	– 1.22 (3.25)
CRN				
FCz	– 1.10 (1.24)	– 0.99 (1.45)	– 0.90 (1.75)	– 0.57 (1.84)
Fz	– 1.90 (1.27)	– 1.62 (1.20)	– 1.54 (1.87)	– 1.17 (2.05)
∆ERN				
FCz	– 0.98 (2.14)	– 2.17 (2.67)	– 1.64 (2.59)	– 2.19 (2.45)
Fz	0.43 (1.95)	– 0.97 (2.31)	– 0.32 (1.58)	– 0.05 (2.50)

ERN = error-related negativity, CRN = correct response negativity, ∆ERN = difference between error-response negativity and correct-response negativity.

#### Correlational analyses

To investigate whether the treatment-related changes in ERN and ∆ERN were related to changes in the SART indices or to changes in self-reported depression (BDI II), anxiety (GAD 7), or levels of formal mindfulness practice, we computed correlations between the residualized change scores. None of these correlations reached significance, all *p*s > .10.

## Discussion

In line with our hypothesis, we found that, in the absence of task conditions that induce increased salience of errors and threat, chronically depressed patients show a substantially reduced ERN relative to healthy controls. The present study was one of the largest to date, in terms of numbers of depressed patients, and given that the observed effect was highly significant and of considerable size, the deficits identified should be considered a robust finding. The finding is in line with other recent research suggesting that a blunted ERN is a characteristic of patients who show vulnerability for more persistent forms of depression (Weinberg, Liu, & Shankman, 2016), and it is broadly consistent with evidence suggesting that reward-related abnormalities become exacerbated with increased disease burden (Hall et al., 2014). The fact that deficits were observable not only in frontocentral but also in frontal regions corresponds with previous research indicating that depressed patients show changes in the distribution of the global electrical field associated with errors (Aarts et al., 2013), which has particularly implicated the role of medial prefrontal generators of the ERN. However, the present data do not allow any strong topographical claims in this context, given that we detected no significant Group × Electrode interaction on the ERN.

Although research so far has shown that aberrations in ERN remain even when treatments have brought symptomatic relief (Hajcak et al., 2008; Riesel et al., 2014; Schrijvers et al., 2009), the present data suggest a potential for reversibility. Although we did not find evidence for differential treatment effects in frontocentral regions, our results indicated significant increases in the ERN in frontal regions following training in mindfulness meditation, whereas there was no significant change in these regions in those who had received a resting control. These findings suggest that mindfulness training may be helpful in reducing deficits in activity in the more frontal regions contributing to the generation of the ERN with differences between effects in frontocentral and frontal sites gradual rather than significant in nature.

Our findings thus provide preliminary evidence that the ERN can be changed through mental training that is specifically aimed at the functions associated with the ERN, and they extend previous research that has shown increased ERN in healthy individuals with a regular meditation practice (Teper & Inzlicht, 2013). Importantly, the mindfulness training seems to have compensated exactly for those deficits that characterized depressed patients in comparison to healthy controls at baseline. This is potentially significant, given the large body of previous research that has demonstrated downstream consequences of ERN aberrations (Botvinick et al., 2001) and the role of such deficits in vulnerability to psychopathology (Manoach & Agam, 2013; Olvet & Hajcak, 2008). The fact that changes in ERN magnitude were independent of changes in the symptoms supports the view that the observed effects did not simply occur as an epiphenomenon of symptom reductions, but are likely to be attributable to the particular characteristics of the intervention, rather than simply representing an indicator of the severity of depression. Changes in these variables may occur on different timescales and may not necessarily correlate at all points in time.

Mindfulness practice combines attentional training with the cultivation of an open and welcoming stance toward current experience, and it may thus influence the ERN through both modulating affective components and increasing the underlying cognitive capacities. Consistent with the former possibility, evidence has emerged that inductions of an attitudinal stance of mindfulness, leading to changes in the way that individuals relate to their emotional experience, can have immediate effects on ERN magnitude (Saunders, Rodrigo, & Inzlicht, 2016; see also Hobson, Saunders, Al-Khindi, & Inzlicht, 2014). When assessed in the absence of conditions that serve to modulate affective components, the ERN seems to be more difficult to change. A study in which participants simply engaged in a brief focused attention meditation did not have any effects on subsequently assessed ERN magnitude (Larson, Steffen, & Primosch, 2013), thus pointing toward the importance of repeated practice. A previous imaging study reported changes in white matter integrity in the anterior cingulate cortex, which is assumed to be one of the main neural generators of the ERN, after 11 h of practice (Tang et al., 2010), a level of practice that is almost identical to that in our study. Given the suggested role of aberrations in ERN as an endophenotype for psychopathology and previous reports of resistance to treatment, the present findings are notable in suggesting that mindfulness training may offer a potential pathway to counter an otherwise relatively stable vulnerability.

Interpretations of the present findings will need to take into account a number of limitations and caveats. First, the tests of treatment effects were based on a small sample of patients and are therefore in need of replication. Second, although the present findings are based on patients with chronic depression, it is not possible from our study to strictly conclude that the findings are specific to this group, which would have required the inclusion of a depressed control group without a chronic course of the disorder. Third, it remains unclear from the present study to what degree the observed changes in ERN would continue to be present following the end of the intervention. To address this question, future research will have to include follow-up assessments after the end of the intervention. Fourth, although the changes in ERN were unrelated to changes in the symptoms, it is possible that the effects might have been driven by other, unmeasured factors. For example, it has been shown that incentive conditions can enhance the ERN (Moser et al., 2013), and our differential effects thus could potentially be due to differences in motivation to take part in the assessment after the intervention. Fifth, since both the mindfulness and control groups showed reductions in ERN amplitude and symptoms over time, it remains possible that part of the changes observed were due simply to the effects of time rather than to any specific effects of the treatments.

In summary, the present findings demonstrate significant ERN deficits in chronically depressed patients. In contrast to previous treatment research on the ERN, but consistent with previous studies demonstrating effects of meditation on the structure and function of the medial frontal cortex (Grant, Courtemanche, Duerden, Duncan, & Rainville, 2010; Hölzel et al., 2011; Tang et al., 2010), we found preliminary evidence that training in mindfulness meditation can help to effectively reverse these deficits and significantly increase ERN magnitude. The latter findings warrant a larger-scale confirmatory replication.

### Author note

T.B. is now at the University of Exeter, Sir Henry Wellcome Building for Mood Disorders Research, Perry Road, Exeter, UK EX4 4QG. J.M.H. is now at the Max-Planck Institute for Human Cognitive and Brain Sciences, Max-Planck Research Group for Neuroanatomy and Connectivity, Stephanstrasse 1a, 04103 Leipzig, Germany. M.G. is now at Charité University Medicine Berlin, Clinic for Psychiatry and Psychotherapy, Hindenburgdamm 30, 12203 Berlin. This research was funded by German Research Foundation Grant No. BA2255 3-1, awarded to T.B. T.B. was also supported by a Heisenberg Fellowship from the German Research Foundation (BA2255 2-1). The funders had no role in the study design; in the collection, analysis, and interpretation of the data; in the writing of the report; or in the decision to submit the article. We thank our participants for giving their time to take part in the study. The study is registered at ClinicalTrials.gov (NCT02801513). Data and significant results will be made available upon request to the corresponding author.
